# Prevalence of metabolic syndrome-related disorders in a large adult population in Turkey

**DOI:** 10.1186/1471-2458-6-92

**Published:** 2006-04-10

**Authors:** S Yavuz Sanisoglu, Cagatay Oktenli, Adnan Hasimi, Mehmet Yokusoglu, Mehmet Ugurlu

**Affiliations:** 1 Department of Biostatistics, Gülhane Military Medical Academy, Ankara, Turkey; 2 Department of Internal Medicine, Gülhane Military Medical Academy, Ankara, Turkey; 3 Department of Biochemistry and Clinical Biochemistry, Gülhane Military Medical Academy, Ankara, Turkey; 4 Department of Cardiology, Gülhane Military Medical Academy, Ankara, Turkey; 5 Turkish Ministry of Health, Ankara, Turkey

## Abstract

**Background:**

There are few existing large population studies on the epidemiology of metabolic syndrome-related disorders of Turkey. The purpose of this study was to assess the prevalence of metabolic syndrome-related disorders in the Turkish adult population, to address sex, age, educational and geographical differences, and to examine blood pressure, body mass index, fasting blood glucose and serum lipids in Turkey.

**Methods:**

This study was executed under the population study "The Healthy Nutrition for Healthy Heart Study" conducted between December 2000 and December 2002 by the Health Ministry of Turkey. Overall, 15,468 Caucasian inhabitants aged over 30 were recruited in 14 centers in the seven main different regions of Turkey. The data were analyzed with the Students' t, ANOVA or Chi-Square tests.

**Results:**

Overall, more than one-third (35.08 %) of the participants was obese. The hypertensive people ratio in the population was 13.66 %, while these ratios for DM and metabolic syndrome were 4.16 % and 17.91 %, respectively. The prevalence of hypertension, metabolic syndrome and obesity were higher in females than males, whereas diabetes mellitus was higher in males than females. The prevalence of metabolic syndrome and related disorders were found to be significantly different across educational attainments for both men and women. The prevalence of hypertension increased with age, while it was remarkable that in the age group of 60–69 years, prevalence of diabetes mellitus and metabolic syndrome reached a peak value and than decreased. For obesity, the peak prevalence occurred in the 50–59 year old group. The prevalence of metabolic syndrome and related disorders were found to be significantly different according to geographical region.

**Conclusion:**

In conclusion, high prevalence of obesity and metabolic syndrome, particularly among women, is one of the major public health problems in Turkey. Interestingly, obesity prevalence is relatively high, but the prevalence of hypertension and hypercholesterolemia is relatively low in Turkish people. Future studies may focus on elucidating the reasons behind this controversy. Our findings may be helpful in formulating public health policy and prevention strategies on future health in Turkey.

## Background

Turkey, with its large land area, more than 70 million people, and growing economy, resembles a bridge in geographical aspects between Europe and Asia. There are few existing large population studies on the epidemiology of metabolic syndrome-related disorders of Turkey. Turkish Adults Risk Factor Study (TEKHARF) was the first study, representing the whole country, conducted by the Turkish Cardiology Association [1].

The etiology, prevention, and treatment of the metabolic syndrome currently are the focus of the intense research activities. The combination of abdominal obesity, hypertension, dyslipidemia, hyperglycemia, and insulin resistance or type 2 diabetes mellitus defines the metabolic syndrome [2]. The metabolic syndrome is said to consist of a cluster of metabolic risk factors, including dyslipidemia, impaired carbohydrate metabolism, obesity, and high blood pressure [3,4]. In 1999, the WHO published the first official definition of the metabolic syndrome [5]. The new IDF definition of the metabolic syndrome addresses both clinical and research needs and provides an accessible, diagnostic tool that is suitable for use in populations worldwide [6]. The pathogenesis of metabolic syndrome is still unclear, although some environmental factors, coupled with still largely unknown genetic factors, clearly interact to produce the syndrome [7,8]. Previous reports using population-based data from the Third National Health and Nutrition Examination Survey have estimated the age-adjusted prevalence of metabolic syndrome to be 21.8% among adults in the United States [9-11].

The purpose of this study was to assess the prevalence of the metabolic syndrome-related disorders in the Turkish adult population of more than 15,000 persons, to address sex, age, educational and geographical differences, and to examine blood pressure, BMI, fasting blood glucose and serum lipids in Turkey.

## Methods

**Figure 1:**
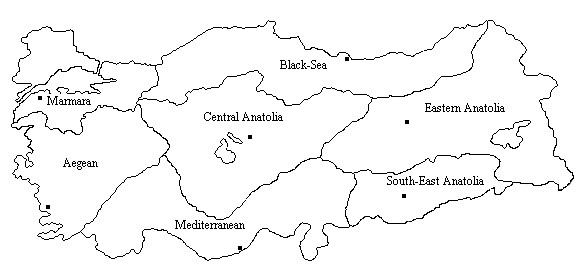
Main Geographical Regions and Location of the Study Centers.

This study was executed under the population study "The Healthy Nutrition for Healthy Heart Study" conducted between December 2000 and December 2002 by the Health Ministry of Turkey. Overall, 15,468 Caucasian inhabitants aged over 30 were recruited in 14 centers in the seven main different regions of Turkey that would represent whole Turkish People in terms of living conditions and geographic background (Figure 1). Marmara, Aegean and Mediterranean regions are in the western part of Turkey, while Eastern and South-Eastern Anatolia are in the eastern part. Black Sea region is in the northern side. The number of people for each center was calculated by using stratified sampling technique. In the selection of the participants, we used random number table. In order to cover the whole population of Turkey, men and women were randomly selected from urban and rural areas from all regions. Sample size for the study was calculated by allowing for 0.25% error in prevalence with 95% confidence interval (CI). At the end of the study, we reached 15,468 people. The response rate was 87.3 %. Sample sizes for each health center were calculated using the census of the year 2000 by stratified sampling method [12]. Data form was developed after the execution of a pilot study. All socio-demographic data (age, gender, and educational level), and medical history were obtained through a medical staff by using a standardized questionnaire in the study centers. Educational attainment was categorized as illiterate, literate only, elementary education, high school graduate or a university education. Completion of the questionnaire was considered to imply informed consent. The study was carried out in accordance with the guidelines of the Helsinki Declaration of Human Studies.

Study participants were studied in six separate age groups, namely those aged 30–39, 40–49, 50–59, 60–69, 70–79 and those aged older than 80. The survey was representatively stratified for sex, age, and for rural-urban distribution.

Seven measures representing the metabolic syndrome were obtained, including fasting blood glucose, BMI, HDL-C and Low-density lipoprotein cholesterol (LDL-C), triglycerides, systolic blood pressure, and diastolic blood pressure. At the baseline examination, blood samples were taken after a minimum 6-hour fast. Serum was separated on-site within 30 minutes of venipuncture, stored at -4°C, and analyzed within 24 hours of venipuncture. Determination of routine biochemical parameters was performed with standard techniques by using an autoanalyser. Values for each person were calculated by Friedewald's formula and LDL-C values >10.36 mmol/l were not taken into account.

Because waist circumference was not determined in this study, we defined the metabolic syndrome using BMI (≥ 30 kg/m^2 ^for both men and women) instead of waist circumference. BMI was calculated as the ratio of body weight to square of body height (kg/m^2^). Obesity was defined based on BMI 30 kg/m^2^. Blood pressure was calculated as the average of three measurements taken under standardized conditions in a supine position with a sphygmomanometer. Diabetes mellitus was defined as a fasting blood glucose level = 7 mmol/l, or reported use of diabetes medication. The IDF definition was used for the diagnosis of the metabolic syndrome [6].

A total of data were analyzed by StatsDirect (Ver 2.2.0, Stats Direct Ltd, UK) and SPSS 11.5 (SPSS Inc., Chicago, Il., USA) software. Descriptives were shown either the percentages or mean ± SD for categorical and continuous variables, respectively. In order to minimize the effects of the outliers we calculated the 5% trimmed means. Parameters of two group of people were compared by "Independent samples t test". "Analysis of variance (ANOVA test)" was used to compare the parameters of more than two groups. Relations among the categorical parameters were investigated by "Chi-square test". *p*values less than or equal to 0.05 were considered as statistically significant.

## Results

A total of 2096 (13.6 %) were from Marmara, 1555 (10.1 %) were from Eastern Anatolia, 2541 (16.4 %) were from Southeast Anatolia, 2554 (16.5 %) were from Mediterranean, 2519 (16.3%) were from Aegean, 2190 (14.2 %) were from Black Sea, and 2013 (13.0 %) were from Central Anatolia.

Based on self-report, 20.3% were illiterate (n = 3123), 18.0 % were literate only (n = 2766), 41.2% of the people (n = 6334) were elementary education, 13.1 % were high school graduate (n = 2015), and 7.4% were university educated (n = 1136).

Overall, more than one-third (35.08 %) of the participants was obese. The hypertensive people ratio in the population was 13.66 %, while these ratios for DM and metabolic syndrome were 4.16 % and 27.38 %, respectively.

The prevalence of hypertension, metabolic syndrome and obesity were higher in females than males, whereas diabetes mellitus was higher in males than females (Table 1). There were no statistically significant difference between the urban and rural areas with respect to metabolic syndrome and related disorders such as hypertension, diabetes mellitus, and obesity (data not shown). The prevalence of metabolic syndrome and related disorders were found to be significantly different across educational attainments for both men and women (Table 1). Obese men and women tended to have lower educational attainment (Table 1). The prevalence of hypertension increased with age, while it was remarkable that in the age group of 60–69 years, prevalence of diabetes mellitus and metabolic syndrome reached a peak value and than decreased (Table 1). For obesity, the peak prevalence occurred in the 50–59 year old group. The prevalence of metabolic syndrome and related disorders were found to be significantly different according to geographical region (Table 1).

The distribution across regions of biochemical parameters by geographical region, gender and age groups is presented in the Table 2 and 3. The differences of biochemical parameters among the geographical regions were significantly different (Table 2), while they, except HDL-C, were significantly different among the age groups (Table 3). When the data compared according to gender, we found that total cholesterol and LDL-C in the age groups of 30–39, 50–59 and 60–69, total cholesterol and triglyceride in the 70–79 age group, and triglyceride in 40–49 age group were significantly different (Table 3).

Mean total cholesterol level of our study population was 4.92 mmol/l (4.91 mmol/l in men and 4.92 mmol/l in women). Hypercholesterolemic levels were recorded in 29.89% in men, 28.35 in women in the present study. Mean LDL-C level was 2.92 mmol/l (2.96 mmol/l in men, 2.90 mmol/l in women). High values of LDL-C were found 29.89% in men and 28.35% in women in our study. Mean concentrations of HDL-C, were 1.10 mmol/l (1.10 mmol/l in men and women). In the current study, lower HDL-C concentrations were found 25.16% in men and 32.39% in women. In our study, mean concentrations of triglyceride were 1.76 mmol/l (1.80 mmol/l in men and 1.75 mmol/l in women). In addition, prevalence of hypertriglyceridemia in our study was 35.54% (40.72% in men and 33.23% in women).

## Discussion

This study is one of the largest population-based studies of metabolic syndrome-related disorders ever conducted in Turkey. The prevalence of metabolic syndrome was 10.09 and 27.33 % in men and women, respectively. Onat *et al*. reported a higher prevalence of the metabolic syndrome in Turkish adults (27% in men and 38.6% in women) than our findings [13]. In the National Health and Nutrition Examination Survey sample in U.S. adults [11], age-adjusted prevalence of the metabolic syndrome was 24.0% and 23.4% in men and women, respectively. In the current study, the prevalence of diabetes was determined to be 4.16% (5.22% in men and 3.69% in women). Onat *et al*. reported that prevalence of diabetes in a Turkish population was 3.4% (4.2% for men and 2.8% for women) [13]. In The Turkish Diabetes Epidemiology Study (TURDEP), the prevalence of diabetes was 7.4% (6.2% in men and 8.0% in women) [14]. On the other hand, the overall prevalence of obesity was 35.08 % (21.16% in men and 41.32% in women) in our study. Similar to our results, in a large multicenter nationwide study [1], this prevalence was found to be 25.3% for Turkish males and 44.2% for Turkish females. Conversely, Satman *et al*. reported that the low prevalence of obesity in adults in Turkey was 22.3% (12.9% in men, 29.9% in women) [14]. Finally, the prevalence of hypertension in our study (13.66%) was relatively lower than a previous study (46%) [15].

In the present study, there was a clear age-related increase in the prevalence of the metabolic syndrome in Turkish adult population. The prevalence of metabolic syndrome was the lowest at age group 30–39 (15.34%), while it progressively increased with age until the age group 50–59 (27.98%). Ford *et al*. reported the prevalence of metabolic syndrome increased with age, and 33–45% of subjects over 50 years met the criteria for the metabolic syndrome [11]. In this context, aging may be a risk factor for metabolic syndrome. In many studies, it was also reported that the prevalence of diabetes increased with age [16-20]. Likewise, in the current study, the prevalence of obesity increased markedly from the 30- to 39-year-old age group to the 50- to 59-year-old age group and then decreased. In many studies, it has been reported that prevalence of obesity increases with age [21-23]. This can be explained partly by a decrease in the degree of physical activity by aging [21,24].

The prevalence of diabetes, obesity, hypertension and metabolic syndrome was found to be decreased with high educational levels in our study. The results are in line with studies conducted in Turkey and Europe [21,25-27,31]. In a recent study [25], a clear association was reported between education and blood glucose levels and a higher risk of diabetes associated with lower levels of education. It is known that unhealthy dietary habits as a high fat intake and low fruit and vegetable intake, as well as physical inactivity, are inversely related with educational level [32].

We found that the prevalence of diabetes among the regions was the highest in South-East Anatolia region. A study conducted by Gokcel *et al*. [20] in Adana, a city in South-East Anatolia, reported the diabetes prevalence as 11.6%. On the contrary, as reported previously [33], the lowest prevalence of diabetes was obtained from the participants living in the Eastern Anatolia Region of Turkey. In consistent with our observation, in Central Anatolia, the prevalence of diabetes was reported as 6.4% [19] and 6.9% [34]. Similar to our results, the prevalence of diabetes was 5.2% in Trabzon, a city located in the Black Sea Region. On the other hand, in contrast to a previous report [14], subjects living in the Eastern and South-East Anatolia Region of Turkey had the highest prevalence of obesity in our study. However, consistent with the same report [14], obesity prevalence in Central Anatolia was high. In the Black Sea Region, prevalence of obesity was found to be higher (36.89%) than a previous study (19.2%) [35]. The average values of total cholesterol, LDL-C, and triglyceride were also higher in South-Eastern Anatolia, Eastern Anatolia and Central Anatolia than the other regions. Consumption of foods with high fat and sugar content is also frequent in this area [36]. In our study, the prevalence of obesity in Marmara Region is consistent with a previous report [37] and this region has the lowest obesity prevalence as well as Aegean region. This may be due to participants from these regions consume more vegetables, less carbohydrate; prefer olive oil or corn oil more when cooking [38,39]. The hypertension prevalence among the geographical areas in Turkey is also considerably different. Like a previous report [14], the prevalence was lower in the western region (Mediterranean and Aegean Regions) than the northern region (Black Sea Region). Environmental factors or individual exposures such as salt intake may cause such differences in prevalence among these populations.

In the current study, as in others [40,41], mean total cholesterol, LDL-C and HDL-C level of Turkish adults was lower than the Northern Europe and the Mediterranean populations [42]. The mean concentrations of triglyceride were higher than the Turkish Heart Study [43].

## Conclusion

In conclusion, high prevalence of obesity and metabolic syndrome, particularly among women, is one of the major public health problems in Turkey. Interestingly, obesity prevalence is relatively high, but the prevalence of hypertension and hypercholesterolemia is relatively low in Turkish people. Future studies may focus on elucidating the reasons behind this controversy. Our findings may be helpful in formulating public health policy and prevention strategies on future health in Turkey.

## Competing interests

The author(s) declare that they have no competing interests.

## Authors' contributions

SYS carried out the data organization and performed the statistical analysis. CO participated in the evaluation of data. AH and MY contributed to the study with their knowledge on biochemistry and cardiology, respectively. MU participated in coordination and evaluation of data.

## Note

Table 1 – Prevalence of Metabolic Syndrome and Related Disorders

Table 2 – Distribution and Comparisons Results of the Biochemical Parameters for The Geographical Regions (5% Trimmed Mean) FBG, fasting blood glucose; TC, total cholesterol; TG, triglyceride

Table 3 – Descriptive Statistics of the Parameters According to the Age Groups and Gender (5% Trimmed Mean) F, female; M, male; FBG, fasting blood glucose; TC, total cholesterol; TG, triglyceride

## Pre-publication history

The pre-publication history for this paper can be accessed here:
